# Mortality risk associated with clinical signs of possible serious bacterial infection (PSBI) in young infants in Africa and Asia: protocol for a secondary pooled analysis

**DOI:** 10.1136/bmjopen-2024-097135

**Published:** 2025-06-24

**Authors:** Gary L Darmstadt, Vaishnavi Bhamidi, Khusbu Adhikari, Ivana Marić, Mohammad Shahidul Islam, Shamim Ahmad Qazi, Saifuddin Ahmed, Antoinette Tshefu Kitoto, Fabian Esamai, Adejumoke Idowu Ayede, Ebunoluwa A Adejuyigbe, Robinson D Wammanda, Samir K Saha, Yasir Bin Nisar

**Affiliations:** 1 Department of Pediatrics, Stanford University School of Medicine, Stanford, California, USA; 2 Department of Epidemiology and Population Health, Stanford University School of Medicine, Stanford, California, USA; 3 Department of Computer Science, Stanford University, Stanford, California, USA; 4 Child Health Research Foundation, Dhaka, Bangladesh; 5 Independent Consultant Paediatrician, Geneva, Switzerland; 6 Department of Population, Family and Reproductive Health, Johns Hopkins University, Baltimore, Maryland, USA; 7 University of Kinshasa, Kinshasa, Congo (Democratic Republic of the Congo); 8 Department of Child Health and Paediatrics, Moi University, Eldoret, Uasin Gishu County, Kenya; 9 University of Ibadan College of Medicine, Ibadan, Oyo, Nigeria; 10 Department of Paediatrics and Child Health, Obafemi Awolowo University, Ile-Ife, Osun, Nigeria; 11 Ahmadu Bello University Teaching Hospital, Zaria, Kaduna state, Nigeria, Zaria, Kaduna, Nigeria; 12 Department of Maternal, Newborn, Child and Adolescent Health and Ageing, World Health Organization, Geneve, Switzerland

**Keywords:** Mortality, Clinical Decision-Making, International health services, Protocols & guidelines, Machine Learning, Community child health

## Abstract

**Abstract:**

**Introduction:**

The WHO’s Integrated Management of Childhood Illness (IMCI) in young infants <2 months of age includes the identification and management of signs of possible serious bacterial infection (PSBI). However, equal importance is given to all the PSBI signs, which signal the need for referral and hospital management, except for fast breathing in infants aged 7–59 days, for which outpatient treatment by clinical staff working at a health facility is recommended. Moreover, studies to validate the importance of clinical signs of PSBI have mostly used the need for hospitalisation as the outcome. There is a need to further examine the association of signs of PSBI individually and in combination with risk of mortality and to analyse global data to inform global recommendations.

**Methods and analysis:**

We will create a dataset that integrates data from population-based studies globally with similar designs that have examined the presence of signs of PSBI identified by frontline health workers throughout the young infant period (days 0 to <60) and that have also recorded infant vital status. We will conduct pooled, individual-level analyses of the frequency of identification of signs individually and in combinations and will conduct three types of analyses of association of signs of PSBI with mortality: (1) case fatality, which has been used in a multisite study of mortality risk associated with signs of PSBI in young infants in Africa; (2) Cox regression, which will enable time-varying analysis of exposure in relation to mortality, as has been done in a multisite study in Asia and (3) machine learning analysis, which has not previously been applied to any of the available data.

**Ethics and dissemination:**

All prior studies incorporated into our pooled analysis were approved by the independent local ethics committee/institutional review board (IRB) at each study site in each country, and all study participants provided informed consent. This project was approved by the Stanford University School of Medicine IRB protocol 74456. Study findings will be disseminated through publications in peer-reviewed journals, WHO documents, and presentations at maternal and child health meetings.

**STRENGTHS AND LIMITATIONS OF THIS STUDY**

- Pooling of individual subject data available globally.
- Pooling of population-based studies using common designs for subject enrolment, baseline covariates and assessment of community health worker (CHW)-identified clinical signs of the WHO’s Integrated Management of Childhood Illness singly and in combinations and vital status.
- Use of analytic methods to assess risk for mortality that complement (case-fatality and regression) and extend (machine learning) prior approaches.
- There may have been some variations in the assessments of clinical signs by the CHWs across study sites, although efforts were made to standardise them.

## Introduction

 Neonatal deaths comprise nearly half of all mortality in children before their fifth birthday.^1^ The most common causes of neonatal mortality, based on modelled estimates from global data, are complications of preterm birth (0.88 million, 36.0%); intrapartum-related events (‘birth asphyxia’) (0.58 million, 23.8%) and infections including pneumonia, sepsis and meningitis (0.4 million, 16.4%).^2^ Based on data from 11 surveillance sites in Africa and Asia, the Alliance for Maternal and Newborn Health Improvement (AMANHI) mortality study group found that perinatal asphyxia (40% in South Asia and 35% in sub-Saharan Africa) and severe infections (35% in South Asia and 37% in sub-Saharan Africa) were the most common causes of deaths in the neonatal period, followed by complications of preterm birth (19% in South Asia and 24% in sub-Saharan Africa).^3^ Similar results for rates and cause-specific mortality distributions were found in a 5-site, 3-country study in South Asia (Aetiology of Neonatal Infection in South Asia (ANISA)).^4^

Estimates of the incidence of severe neonatal infections have varied, but they nevertheless emphasise the magnitude of the burden. In 2012, a systematic literature review with meta-analysis estimated that the incidence of possible serious bacterial infection (PSBI) was 7.6%, totalling 6.9 million cases, with a case-fatality rate (CFR) of 9.8% in sub-Saharan Africa, South Asia and Latin America.^5^ A 6-country prospective study in 2010–2013 reported a 12.9% incidence of PSBI in the first 6 weeks after birth and a CFR of 14.0%.^6^

Recognising the need to provide guidelines on the identification and management of infections in young infants, based on several studies of clinical signs of illness associated with a need for hospitalisation of young infants during days 0 to <60,^7^,^13^ the WHO published Integrated Management of Childhood Illness (IMCI) in 2014, which included 7 signs of PSBI indicating the need for referral of sick young infants for hospital care.^14^ These signs were: not able to feed at all or not feeding well, convulsions, severe chest indrawing, high body temperature (38°C or above), low body temperature (less than 35.5°C), movement only when stimulated or no movement at all, or fast breathing (60 breaths per minute or more). In 2015, WHO further recommended outpatient treatment of infants aged 7–59 days who present with isolated fast breathing with oral amoxicillin without a referral,^15^ which was included in the 2019 IMCI chart booklet for young infants.^16^ WHO guidelines currently give equal importance to the other six PSBI signs. However, implementation of IMCI would be further aided by the identification of young infants with signs of illness at a lower risk for mortality who could potentially be treated on an outpatient basis. In addition, using mortality as the outcome in assessing the risk associated with signs of PSBI rather than the need for hospitalisation may enhance policy relevance.

We aimed to identify and pool individual subject data from studies globally that used similar methods to systematically assess infants for signs of PSBI, conducted by community-based frontline health workers, while also recording vital status during the young infant period. We will examine associations of signs individually and in combination with mortality to further define a level of risk for mortality and inform WHO IMCI global guidance for the assessment and management of young infants.

### Methods and analysis

#### Study populations

A literature review in consultation with WHO child health staff was conducted to identify population-based studies which assessed clinical signs of PSBI as defined by WHO^14^,^16^ and recorded the timing of deaths of young infants aged 0 to <60 days. We identified 6 studies which met these criteria, including the Simplified Antibiotic Treatment Trial (SATT) Pakistan,^17 18^ the African Neonatal Sepsis Trial (AFRINEST),^19^,^22^ Aetiology of Neonatal Infection in South Asia (ANISA),^23 24^ a community-based study in Pakistan,^25^ the Projahnmo-2 study in Bangladesh,^12 26 27^ and the National Institute of Child Health and Human Development (NICHD) Global Network’s Maternal and Newborn Health Registry study.^6^ The SATT Pakistan trial used community health workers (CHWs) to assess young infants for signs of PSBI in the community, but infants identified with signs were referred to health clinics and, if eligible, were enrolled in an antibiotic treatment trial and subsequently followed by nurses at the clinic.^18^ Since our study is aimed at assessing the risk of mortality associated with any sign of PSBI, alone or in combination, identified by CHWs and not physicians, and because data on CHW assessments across the entire young infant period were not available on infants enrolled in the SATT Pakistan study (as determined through discussion with the study principal investigators), this study was excluded. Zaidi *et al* evaluated young infants in Karachi, Pakistan, for PSBI identified using an algorithm-based approach that included signs overlapping with current IMCI signs of PSBI.^25^ Among infants identified with PSBI, families of infants who refused hospital admission were consented and enrolled in a three-arm community-based antibiotic treatment trial; however, because reporting on mortality outcomes was limited to these infants and was not population-based, this study was not included in our analysis. The NICHD Global Network’s Maternal and Newborn Health Registry study enrolled infants across seven rural sites in six countries who were assessed for the presence of various signs of serious infections and vital status over the first 6 weeks after birth. However, the variables in the site registries were not designed for the diagnosis of PSBI, and information for identifying infants with PSBI was not uniform across sites; thus, this study was excluded from our analysis.^6^

The two remaining studies (AFRINEST and ANISA) each used CHWs to conduct population-based surveillance to identify pregnancies, births and pregnancy outcomes and collected data through household visits on WHO signs of PSBI in young infants aged 0–59 days (tables1  2). Young infant clinical signs were assessed by similarly trained CHWs or community health extension workers (CHEWs, in the Nigeria site of AFRINEST) at the same time intervals over the first 60 days after birth. Home visits to assess young infants for signs of PSBI were an entry point into antibiotic treatment trials for the AFRINEST study^21 22^ or an observational study of infectious aetiology for ANISA.^24^ The management of infants with signs of PSBI differed across these studies—with randomisation of infants in the AFRINEST^21 22^ trials to various antibiotic treatment regimens, while ANISA^24^ focused on the identification of the aetiology of infection, and staff at the study sites managed infants according to local standard practice—which could have led to differential mortality risk across the studies. It is notable that the young infant mortality rate in the AFRINEST study (9.2 deaths in infants <60 days of age per 1000 live births)^28^ was substantially lower than the neonatal mortality rate for sub-Saharan Africa reported by AMANHI (20.1 per 1000 live births),^3^ which may reflect the treatment provided to infants in the AFRINEST study and may limit generalisability of the results of our analysis. ANISA confirmed young infant deaths and determined the cause of death using verbal autopsy, but AFRINEST did not.

**Table 1:** Summary of studies to be included in pooled analysis of associations between clinical signs of PSBI and mortality of young infants in Africa and Asia

	AFRINEST^19^,^22^	ANISA^23 24^
Study design	Prospective, open-label equivalence RCTs	Prospective observational cohort study
Study years	2011–2013	2010–2015
Study sites	The DRC (Equateur province)Kenya (Busia, Bungoma and Kakamega counties in Western Kenya), Nigeria (Ibadan, Ile-Ife, Zaria)	India (Vellore, Odisha)Bangladesh (Sylhet)Pakistan (Karachi, Matiari)
Study aim	To evaluate simpler antibiotic regimens for the provision of safe and effective treatment of 0- to 59-day-old young infants with signs of clinically severe infection whose families do not accept or cannot access referral-level care. One trial had four arms and evaluated simplified antibiotic regimens for young infants with signs of clinical severe infections.^20^ Another trial had two arms and evaluated whether young infants with fast breathing alone can be safely and effectively treated in the community with oral antibiotics alone.^21^	(1) To establish community-based surveillance to identify cases of PSBI among infants aged 0–59 days and collect specimens for aetiological evaluation; (2) to determine community-acquired aetiology-specific incidence of bacterial and viral infections among infants by using standard and molecular diagnostic tools; (3) to identify the risk factors for community-acquired serious bacterial and viral infections in young infants and (4) to identify clinical features predictive of invasive viral and bacterial infections among ill-appearing young infants in the community.
Frontline health worker training	CHWs were used in DRC and Kenya; CHEWs were used in Nigeria.CHWs and CHEWs were trained by supervisory staff from the study sites. CHEWs had 2–3 years of formal training in government-supported training institutes on basic issues of maternal and child health, child nutrition, treatment of infections, immunisation and environmental health and oral and mental health. CHWs had been trained for a few weeks. Refresher training and standardisation exercises were conducted quarterly.	The study protocol was harmonised across sites, and project staff were trained similarly, using a two-stage training programme. In the first stage, experts from WHO trained the supervisory staff from the sites and provided them with training guides, videos and booklets. These trained personnel then trained CHWs from their respective sites for 15–21 days, with the length depending on the experience of the CHWs, local procedures and capacity. Refresher training was given ad hoc as CHWs were followed by a supervisor.
Pregnancy surveillance	Established a pregnancy surveillance system in all study sites. CHWs and TBAs identified and followed up on pregnancies in DRC and Kenya; TBAs and CHEWs identified and followed up on pregnancies in Nigeria.	CHWs identified MWRA and followed them via bi-monthly home visits to identify pregnancies based on the passage of >2 months since the last menstrual period.CHWs recorded the date of the last menstrual period, facilitated or encouraged accessing antenatal care, built rapport and arranged visits to assess newborn infants immediately after birth.
Birth surveillance	CHWs and TBAs identified births in DRC and Kenya, and TBAs and CHEWs identified births in Nigeria within 24 hours of delivery.	CHW followed up with pregnant women during two scheduled visits, immediately after identification of the pregnancy and at the 29th week of pregnancy. The CHWs provided their mobile phone numbers to family members and traditional birth attendants so that they could inform them about the delivery. The CHWs phoned the family every other day starting in the 37th week of pregnancy to enquire whether the mother had delivered.
Young infant inclusion criteria in PSBI surveillance	Liveborn infants in the study area who were visited at least twice by CHWs/CHEWs.	MWRA in the study area gave consent and gave birth to a liveborn infant that a CHW visited within 7 days of birth.
Young infant exclusion criteria in PSBI surveillance	Moved away for delivery or died before follow-up by CHWs/CHEWs after birth.	Infants born live to MWRA in the study area but were not reached by a CHW within 7 days of birth.
Baseline data collection	CHWs collected data using standardised questionnaires during pregnancy and home visits after childbirth. The first visit was as soon as the pregnancy was confirmed, and the second was 2 months before the expected delivery date. At the first home visit after the birth of liveborn infants, CHWs/CHEWs recorded pregnancy outcomes, date of birth, place of birth, birth attendant, postpartum status of the mother, participant demographics, home environment, birth weight, postpartum neonatal characteristics at birth, history of any complaints and examination of the baby to identify any signs of illness. CHW/CHEWs recorded maternal deaths, miscarriages, stillbirths and infant deaths in the first 2 months after birth.	CHWs collected data using standardised questionnaires during pregnancy and the first home visit of liveborn infants on participant demographics, home environment, pregnancy and birth history, postpartum maternal characteristics and neonatal characteristics at birth.
Clinical sign assessment (see table 2 for definitions of signs)	CHWs in DRC and Kenya and CHEWs in Nigeria visited infants at home on days 0, 2, 6, 13, 20, 27, 34, 41, 48 and 59. They identified sick young infants with PSBI signs and referred them to registered nurses at the health centres for further assessment and management. All infants continued to be assessed for all signs of PSBI at the community level by the CHWs and CHEWs, whether or not they were enrolled in a treatment trial.	CHWs made 10 scheduled home visits over the first 60 days after birth, ideally on days 0, 2, 6, 13, 20, 27, 34, 41, 48 and 59.
Management of infants	Infants identified with >1 sign of PSBI and enrolled in antibiotic treatment trials were managed according to trial protocols.^19^,^22^	Infants were managed according to standard care at the study sites.^23 24^ Blood and respiratory cultures were obtained from infants with signs of PSBI to identify the aetiology of infections.
Mortality assessment	The study outcome was the status of the child as alive or dead (all-cause) recorded by CHWs/CHEWs during home visits to assess infants for signs of PSBI.	CHWs assessed vital status at each home visit. Death cases were referred to supervisors of the verbal autopsy study, who conducted a verbal autopsy to determine the cause of death.
Young infants enrolled (N)	84 759 infants	63 017 infants
Young infant deaths (N)	587 deaths	1635 deaths

AFRINEST, African Neonatal Sepsis Trial; ANISA, Aetiology of Neonatal Infections in South Asia; CHEW, community health extension worker; CHW, community health worker; DRC, Democratic Republic of Congo; MWRA, married women of reproductive age; PSBI, possible serious bacterial infection; RCT, randomised controlled trial; TBA, traditional birth attendant.

**Table 2:** Clinical signs of PSBI assessed in the studies included in pooled analysis

PSBI sign	AFRINEST	ANISA
Classification	Young infants in the community were assessed at each home visit by CHWs/CHEWs for all seven signs of PSBI.Young infants with signs of critical illness (inability to feed according to the mother, reported convulsions, no movement at all) were referred to a higher-level hospital for appropriate management. Those who refused referral to a hospital were treated with injectable antibiotics that day and advised for referral again on subsequent days. If they still did not accept the referral, they were treated with injectable antibiotics.Young infants with only fast breathing (infants aged 0–59 days with a respiratory rate >60 breaths per minute) were enrolled and randomised in the fast-breathing AFRINEST trial after the parents refused referral to a higher-level health facility/hospital for management and consented to be enrolled in the RCT.^20 21^Young infants with 1 or more of 5 signs of clinical severe infection (stopped feeding well (observed by a nurse at the health facility), high body temperature (axillary temperature >38°C), low body temperature (axillary temperature <35.5°C), severe chest indrawing and movement only on stimulation) were enrolled and randomised in the clinical severe infection AFRINEST trial after the parents refused referral to a higher-level health facility/hospital for management and consented to be enrolled in the RCT.^19 22^Young infants with signs of local infection (ie, pus in the umbilicus, skin or eye) were treated by a nurse at the health facility according to the WHO guidelines.	Any one sign of PSBI was defined as a case of PSBI.
Fast breathing	Respiratory rate >60 breaths per minute in 0- to <69-day-old infants	Respiratory rate ≥60 breaths per minute
Severe chest indrawing	Severe chest indrawing	Severe chest indrawing: Inward movement of the lower chest wall when the child breathes in
Fever	“High body temperature”: Axillary temperature >38.0°C	Axillary temperature ≥38°C
Hypothermia	“Low body temperature”: Axillary temperature <35.5°C	Axillary temperature <35.5°C
No movement	Movement only on stimulation or no movement at all	No movement or movement only on stimulation
Convulsions	Reported or observed convulsions	Convulsions (reported or observed)
Poor feeding	Stopped feeding well or unable to feed, according to the mother; confirmed by observation	The child is not able to feed at all or stopped feeding well (parental report and observed)

AFRINEST, African Neonatal Sepsis Trials; ANISA, Aetiology of Neonatal Infections in South Asia; CHEW, community health extension worker; CHW, community health worker; PSBI, possible serious bacterial infection; RCT, randomised controlled trial.

### Study design

Given the similarities between the AFRINEST and ANISA studies, we will construct an analytical dataset which allows for pooling the data at an individual level while controlling for study and study sites. The assembly of the dataset began in October 2024, and the analysis will extend to December 2025. We will extract information from each study on baseline demographic and maternal and young infant characteristics. We will also extract temporal data on the presence of clinical signs and young infant mortality. The Strengthening the Reporting of Observational Studies in Epidemiology (STROBE) guidelines for reporting will be followed, with consideration given to Strengthening the Reporting of Observational Studies in Epidemiology for Newborn Infection (STROBE NI),^29^ as these will be observational data.

### Data analysis

#### Exposures and outcomes

We will identify baseline covariates that are common across the studies for use in adjusting regression analyses of risk for mortality and for inclusion in machine learning (ML) models. The study’s exposure is the presence of any of the seven clinical signs of PSBI. The study outcome is all-cause young infant mortality.

#### Analysis

Descriptive analyses will be performed to examine the frequency of visits where each of the signs of PSBI was found alone, as well as where signs were found in combinations. Three types of analyses of risk for mortality will be performed: case fatality, Cox regression and ML. A sensitivity analysis of mortality risk will be carried out at the study (ANISA and AFRINEST) and site levels. When infants were hospitalised, CHWs did not continue to visit the patients and document PSBI signs while the infants were in the hospital; however, CHWs did continue to assess these infants according to the standard visit schedule after discharge from the hospital, and these infants will be retained in the analysis. In ANISA, 4% of infants were hospitalised, while in AFRINEST, 5% of infants were hospitalised. Due to lack of information on the individual identification of CHW assessments, interrater reliability among CHWs cannot be determined; however, CHWs were regularly standardised for the assessment of clinical signs, as described previously.^30 31^

##### Case fatality analysis

AFRINEST data were initially analysed using case fatality analysis, as described previously.^28^ This analysis has the advantage of simplicity of interpretation but lacks consideration of the time-varying risk of mortality. We will use data from the AFRINEST and ANISA studies for infants up to 2 months of age with at least one visit to assess the presence of clinical signs and the outcome following that visit. We will assume infants were alive for all visits until and unless a death is recorded. The case fatality rate will be calculated by sign or a combination of signs as the percentage of young infants who die within 7 days after showing that sign or a combination of signs of PSBI, divided by the total number of young infants who had shown that same sign or a combination of signs during their previous (reference) visit. We specify a 7-day window period to avoid the risk of misclassification or recall bias when no visit data were available for the extended period.

### Cox regression analysis

ANISA data were initially analysed using time-to-event Cox hazards regression models, as described previously.^32^ This same approach will be applied to the pooled data across the AFRINEST and ANISA studies. We will investigate the association between finding a sign of PSBI from the first visit to the end of 60 days after birth and the risk of all-cause mortality compared with not finding such a sign. Infants who were never visited or who died before a CHW visit will be left censored, and exposure periods of unknown status pertaining to the presence of signs of PSBI before the first visit will be eliminated. The period of risk for mortality will be defined as extending for 7 days after finding a sign of PSBI; a sensitivity analysis will be performed using a 14-day period of risk for mortality. We previously showed that for the ANISA data, results were similar for these two periods of risk for mortality.^32^ We will also calculate Kaplan-Meier probabilities of mortality over the young infant period. We will use Cox regression analysis to examine patterns of CHW identification of each sign as follows: a single sign found alone, the sign found with at least one other sign or any sign(s) found other than the particular sign compared with not finding any sign in association with all-cause mortality. Cox regression will also be used to examine the association between the presence of clinical signs and the occurrence of mortality during time frames of the first CHW visit to the end of day 3 (days 0 to <3), the first visit to the end of day 7 (days 0 to <7) and day 7 to the end of the 60th day (days 0 to <60). Analyses will be adjusted for covariates that are in common across all datasets.

### Machine learning analysis

ML models will be trained to predict the risk of death for infants with signs of PSBI. The presence/absence of PSBI signs recorded over multiple visits, as well as available clinical and treatment data, will be used as input features. See supplemental table S1 (in online supplemental file 1) for a preliminary list of available predictors that are common to the ANISA and AFRINEST studies (which includes all predictors in AFRINEST) and supplemental table S2 (in online supplemental file 1) for a list of predictors available in the ANISA dataset. We will use two approaches. First, multivariate modelling will be applied to obtain precise contributions of each sign and combinations of signs to the mortality rate. In this approach, models will be built by applying standard ML models, specifically Elastic Net, XGBoost and Stabl.^33^,^35^ Presence/absence of PSBI signs at different times will be treated as separate features, and the model will predict the risk of death on a specific day given the signs on that day and in the previous 6 days.

Three models will be analysed, each specifying how the risk of death depends on PSBI signs with increasing levels of detail. We denote the predicted (dependent) variable as Y∈{0,1}

where


$$Y=\left\{ \begin{array}{l} 1,\;if\;infant\;dies\;in\;the\;7-day\;window\\ 0,\;otherwise \end{array}\right.$$

In our first (simplest) model 1, we will assume a standard logistic model for the probability of infant death given the signs in the previous 7 days.^36^ We include interaction terms between two different signs to quantify the effect of the presence of two signs on the probability of infant death. The probability that an infant dies given signs in the previous 7 days is then given by


$$\log\left(\frac{P\left(Y=1|X_{1},\ldots ,X_{p}\right)}{P\left(Y=0|X_{1},\ldots ,X_{p}\right)}\right)=\beta_{0}+\sum_{i=1}^{7}\beta_{i}X_{i}+\sum_{i=1}^{7}\sum_{j=2,\;j>i}^{7}\beta_{ij}X_{i}X_{j}+\sum_{i=8}^{p}\beta_{i}X_{i}$$

where input features $X_{i},i=1,\ldots ,7$are given by


$$X_{i}=\left\{ \begin{array}{l} 1,\;if\;sign\;i\;is\;present\;at\;any\;time\;during\;7\;days\\ 0,\;otherwise \end{array}\right.$$

and $X_{i},i=8,\ldots ,p$ are input features for each of the baseline covariates.

The output of the ML model will provide adjusted ORs for the risk of death for each sign and the interaction of signs. ORs for each sign *i* will be obtained as: $OR_{i}=e^{\beta_{i}}$. A limitation of model 1 is that the risk of death depends only on the presence/absence of each of the signs at any time during the 7-day window but does not account for the time lag between the sign and the outcome. Consequently, the probability in model 1 will be the same for two infants that died on different days but had the same signs.

In our second model, we will capture the day in the 7-day window when the death occurred, as follows. For each infant indexed by b that died on day $k_{b}\in \left\{0,\ldots ,6\right\},$ we define $k_{b}$ outcomes $Y_{bk}\in \left\{0,1\right\},k=1,\ldots ,k_{b}$

where


$$\begin{array}{l} Y_{bk}=\left\{ \begin{array}{c} 1,if\;k=k_{b}\\ 0,k§lt;k_{b} \end{array}\right.k=1,\ldots ,\;k_{b}. \end{array}$$

For infants that survived, we have $Y_{bk}=0,\text{for}\;k=0,\ldots ,6$.

We then consider model 2, which, similar to model 1, is a logistic model:


$$\log\left(\frac{P\left(Y_{bk}=1|X_{1},\ldots ,X_{p}\right)}{P\left(Y_{bk}=0|X_{1},\ldots ,X_{p}\right)}\right)=\beta_{0}+\sum_{i=1}^{7}\beta_{i}X_{ik}+\sum_{i=1}^{7}\sum_{j=2,\;j>i}^{7}\beta_{ij}X_{ik}X_{jk}+\sum_{i=8}^{p}\beta_{i}X_{i}$$

where $X_{ik},i=1,\ldots ,7$ denotes the presence or absence of sign *i* at any time before or at day k:


$$X_{ik}=\left\{ \begin{array}{l} 1,\;if\;sign\;i\;is\;present\;at\;any\;day\;before\;day\;k\;or\;at\;day\;k\\ 0,\;otherwise \end{array}\right.$$

In our third model, we capture the timing of each clinical sign. To achieve this, we consider separate features for the presence/absence of PSBI signs for each day:


$$\log\left(\frac{P\left(Y_{k}=1|X_{1},\ldots ,X_{p}\right)}{P\left(Y_{k}=0|X_{1},\ldots ,X_{p}\right)}\right)=\beta_{0}+\beta_{1}^{0}X_{1}^{k}+\beta_{1}^{-1}X_{1}^{k-1}+\ldots +\beta_{1}^{-6}X_{1}^{k-6}+$$

$$\beta_{2}^{0}X_{b}^{k}+\ldots \beta_{b}^{-6}X_{b}^{k-6}+\ldots +\beta_{7}^{-6}X_{7}^{k-6}+\sum_{i=8}^{p}\beta_{i}X_{i}$$

where input features $X_{i}^{k},i=1,\ldots ,7,k=0,\ldots ,6$ are given by


$$X_{ik}=\left\{ \begin{array}{l} 1,\;if\;sign\;i\;is\;present\;at\;any\;day\;before\;day\;k\;or\;at\;day\;k\\ 0,\;otherwise \end{array}\right.$$

In the case of model 3, 1-hot encoding will be used to construct matrix X containing all input features.

We will next perform a survival analysis to estimate the time to death. The standard approach is to apply the Cox proportional hazards model.^37^ In the case where a large number of covariates (and specifically in the case p§gt;N, where N denotes the size of the cohort), the Cox model can experience degenerate behaviour.^38^ To avoid this problem and handle a large number of covariates, we will use a regularised Cox proportional hazards model regularised by Elastic Net.^38^ The Cox model assumes semiparametric hazard in which the hazard for patient i at time t is:


$$h_{i}\left(t\right)=h_{0}\left(t\right)e^{X_{i}^{T}\beta }$$

where $X_{i}=\left(X_{i,1},\ldots ,X_{i,p}\right)$ is a vector of covariates at time t, β is a vector of coefficients of length p and $h_{0}(t)$ is the shared baseline hazard. The vector of coefficients, β, is determined by maximising the partial likelihood


$$L\left(\beta \right)=\prod_{i=1}^{m}\frac{e^{X_{j\left(i\right)}^{T}\beta }}{\sum_{j\in R_{i}}e^{X_{j}^{T}\beta }}$$

which in this case will be subject to the Elastic Net constraint that combines Lasso^39^ and Ridge Regression.^40^

for some threshold *t*. Here, m denotes the total number of time instances in the observation window, j(i) denotes observations failing at time i and $R_{i}$ is a set of indices for which failure occurs at or after time i.

Equivalently, coefficients β will be determined by solving the following:


$$\hat{\beta }={argmax}_{\beta }\left[\frac{2}{N}\left(\sum_{i=1}^{m}X_{j\left(i\right)}^{T}\beta -\log\sum_{j\in R_{i}}e^{X_{j}^{T}\beta }\right)-\lambda P_{\lambda }\left(\beta \right)\right]$$

where


$$P_{\lambda }\left(\beta \right)=\alpha \sum_{i=1}^{p}\left|\beta_{i}\right|+\frac{1}{2}\left(1-\alpha \right)\sum_{i=1}^{p}\beta_{i}^{2}$$

is the Elastic Net penalty. The R package glmnet^41^ will be used for the ML analysis.

The results from these analyses will enable us to gain additional insights, particularly from ML analysis, into combinations of signs of PSBI and potentially other factors that identify young infants with particularly heightened risk for mortality. This information will be taken into account in considering modifications to IMCI for young infants. We will also consider the development of an app—starting, for example, with fast breathing in the first week after birth—to be used by CHWs to guide the management of infants based on information that is useful in predicting risk for mortality.

### Patient and public involvement

Patients and/or the public were not involved in the design or dissemination plans of this research.

### Ethics and dissemination

This pooled analysis will not collect any primary data. All study site protocols were approved by the independent local ethics committee/institutional review board (IRB) at each study site or country, and all study participants gave informed consent. Patient data will be deidentified before inclusion in our analysis. This project was approved by the Stanford University School of Medicine IRB protocol 74456. The analysis was deemed to be exempt from human subjects review by the WHO Ethics Review Committee.

The authors will disseminate the findings of this study through publications in peer-reviewed journals, WHO documents, and presentations at maternal and child health meetings.

## Supplementary material

10.1136/bmjopen-2024-097135 online supplemental file 1
