# Supplementary material for: Mortality risk associated with clinical signs of possible serious bacterial infection (PSBI) in young infants in Africa and Asia: protocol for a secondary pooled analysis
Source: BMJ Open. 2025 Jun 24;15(6):e097135. doi: 10.1136/bmjopen-2024-097135 (PMC12198825; doi:10.1136/bmjopen-2024-097135)
Supplement: online supplemental file 1 [file bmjopen-15-6-s001.docx]

**Supplement**

**Mortality risk associated with clinical signs of possible serious bacterial infection (PSBI) in young infants in Africa and Asia: protocol for a secondary pooled analysis**

**Table S1. Common variables present in both the ANISA and AFRINEST datasets**

|  | **Type** | **Explanation** | **Format of Return** | **Notes** |
| --- | --- | --- | --- | --- |
| General Child Data | Date | Child’s date of birth | MM/DD/YYYY |  |
|  | Date | Child’s date of death | MM/DD/YYYY;  99/99/9999 if child not dead |  |
|  | Categorical | Did the child die? | 0 (No); 1 (Yes) |  |
|  | Categorical | Child sex | 1 (Male); 2 (Female); 999 (Unknown) |  |
|  | Continuous | Child birthweight (grams) | Exact value |  |
|  | Categorical | Z-score less than –3 for birthweight | 0 (No); 1 (Yes) | Calculated from birthweight |
| Birth Context | Continuous | Maternal age (years) | Exact Value;  99 (unknown) | In ANISA study, calculated from maternal month/year of birth and child’s date of birth |
|  | Categorical | Is maternal age <20 years or >35 years? | 0 (No); 1 (Yes); 9 (Unknown) | Calculated from maternal age |
|  | Categorical | Place of delivery | 1(Home);  2 (Hospital/clinic);  3 (Other);  9 (Don't know) |  |
|  | Categorical | Person who delivered the child/ Birth attendant | 1 (Doctor/nurse/midwife);  2 (Traditional birth attendant);  3 (Family members/relatives);  4 (No one);  9 (Other/don't know) |  |
| Clinical Sign Information at Each CHW Visit | Categorical | Respiratory rate > 60 breaths/min | 0 (No); 1 (Yes) |  |
|  | Categorical | Severe chest indrawing | 0 (No); 1 (Yes) |  |
|  | Categorical | Hypothermia (< 35 C) | 0 (No); 1 (Yes) |  |
|  | Categorical | Fever (>38 C) | 0 (No); 1 (Yes) |  |
|  | Categorical | Movement only on stimulation/no movement | 0 (No); 1 (Yes) |  |
|  | Categorical | Poor feeding/no feeding | 0 (No); 1 (Yes) |  |
|  | Categorical | Convulsions (observed and reported) | 0 (No); 1 (Yes) |  |

** All included variables within the AFRINEST CHW dataset are also found in the ANISA CHW dataset

**Table S2. Variables included in the ANISA dataset but which are not present in the AFRINEST dataset**

|  | **Type** | **Explanation** | **Format of Return** | **Notes** |
| --- | --- | --- | --- | --- |
| Child Information at Birth | Categorical | Breath condition of the infant right after birth | 1 (Breathed normally);  2 (Breathed slowly);  3 (Gasping);  4 (Did not breath);  9 (Don’t know) |  |
|  | Continuous | Timing of breathing and/or crying after birth (minutes) | Exact value;  999 (unknown) |  |
|  | Categorical | Any measures taken to get the baby to breathe? | 0 (No); 1 (Yes); 9 (Unknown) |  |
|  | Categorical | Caregiver of the baby right after birth | 1 (Mother herself);  2 (Female family member);  3 (Traditional birth attendant);  4 (Health worker);  5 (Doctor/ nurse);  6 (Male family member);  6 (No one);  9 (Other/don't know) |  |
|  | Categorical | Caregiver washed hands before handling newborn | 0 (No); 1 (Yes); 9 (Unknown) |  |
|  | Continuous | Timing of wiping/drying the newborn (minutes) | Exact value;  00 (Immediately after birth);  90 (>90 mins);  98 (Not wiped/dried);  999 (Unknown) |  |
|  | Categorical | Type of cloth used to wipe/dry the baby | 1 (New);  2 (Used but washed);  3 (Used but not washed);  9 (Unknown) |  |
|  | Continuous | Timing of wrapping of the newborn just after birth | Exact value;  00 (Immediately after birth);  90 (>90 mins);  98 (Not wrapped);  999 (Unknown) |  |
|  | Categorical | Type of cloth used to wrap the baby | 1 (New);  2 (Used but washed);  3 (Used but not washed);  9 (Unknown) |  |
|  | Categorical | Instrument used to cut the umbilical cord | 1 (New blade from delivery kit);  2 (Other new blade);  3 (Used blade/used knife);  4 (Scissors);  5 (Bamboo strips);  9 (Other/ Don’t know); |  |
|  | Categorical | Instrument boiled before used to cut the cord | 0 (No); 1 (Yes); 9 (Unknown) |  |
|  | Categorical | Material used to tie the umbilical cord | 1 (Thread from delivery kit);  2 (Boiled thread);  3 (Other un boiled thread);  4 (Thread brought by doctor);  5 (Clip);  6 (Tongs);  9 (Other/ Don’t know); |  |
|  | Categorical | Anything applied to the umbilical stump immediately after cutting the cord? | 0 (No); 1 (Yes); 9 (Unknown) |  |
|  | Categorical | Newborn given a massage after birth? | 0 (No); 1 (Yes); 9 (Unknown) |  |
|  | Categorical | Materials used for massage | 1 (Mustard oil);  2 (Coconut oil);  3 (Bukwa);  4 (Olive oil);  5 (Sunflower oil);  6 (Ginger);  9 (Other/ Don’t know) |  |
|  | Categorical | Breastfeeding initiated after birth? | 0 (No); 1 (Yes); 9 (Unknown) |  |
|  | Categorical | Timing after birth to initiate breast feeding (minutes) | Exact value;  00 (Immediately after birth);  90 (>90 mins);  98 (Not initiated);  999 (Unknown) |  |
|  | Categorical | Baby is exclusively breastfeeding for the first 3 days? | 0 (No); 1 (Yes); 9 (Unknown) |  |
|  | Categorical | Liquids given to baby other than breast milk in the first 3 days | 1 (Plain water);  2 (Sugar water);  3 (Formula);  4 (Tea);  9 (Other) |  |
| Parental Data | Categorical | Mother has ever had any live birth before this pregnancy | 0 (No); 1 (Yes); 9 (Unknown) |  |
|  | Continuous | Total number of children mother has given birth to in her lifetime | Exact value |  |
|  | Continuous | Total number of mother’s babies that died before they could complete one month | Exact value |  |
|  | Continuous | Measurement of Mid-Upper Arm Circumference (MUAC) of Mother (cm) | Exact Value;  99 (unknown) |  |
|  | Categorical | Religion of the mother | 1 (Hindu);  2 (Muslim);  3 (Christian);  4 (Sikh);  5 (Buddhist);  6 (Jain);  7 (Jewish);  8 (Parsi/Zoroastrian);  9 (Other/Don’t know) |  |
|  | Categorical | Mother’s highest level of education attended | 0 (None); 1 (Primary); 2 (Secondary); 3 (College or higher); 999 (Unknown) |  |
|  | Categorical | Father’s highest level of education attended | 0 (None); 1 (Primary); 2 (Secondary); 3 (College or higher); 999 (Unknown) |  |
|  | Categorical | Mother does any work for income? | 0 (No); 1 (Yes); 9 (Unknown) |  |
|  | Categorical | Whether the woman can go to the health center or hospital | 1 (Can go alone);  2 (Can go with someone);  3 (Cannot go) |  |
| Household Data | Continuous | Total household members | Exact Value;  99 (unknown) |  |
|  | Continuous | Total household members under 5 years | Exact Value;  99 (unknown) |  |
|  | Continuous | Number of sleeping rooms in the household | Exact Value;  99 (unknown) |  |
|  | Categorical | Household’s main material of their roof | 1 (Thatch/palm leaf/reed/grass);  2 (Mud);  3 (Sod/mud and grass mixture);  4 (Wood);  5 (Rustic mat);  6 (Bamboo);  7 (Raw wood planks/ timber/ cardboard);  8 (Plastic/ polyethylene sheeting);  9 (Unburnt brick);  10 (Loosely packed stone);  11 (Metal/GI or iron sheet/tin);  12 (Finished wood);  13 (Calamine/cement fiber);  14 (Asbestos sheet);  15 (Reinforced brick cement/concrete/cement);  16 (Roofing shingles);  17 (Tiles);  18 (Slate);  19 (Burnt brick);  20 (Tent);  99 (Other/ don’t know) |  |
|  | Categorical | Household’s main material of their floor | 1 (Mud/clay/earth/sand);  2 (Dung);  3 (Raw wood planks);  4 (Palm/bamboo);  5 (Brick with no lime/cement);  5 (Rough stone with no lime/cement);  6 (Parquet/polished wood);  7 (Vinyl/asphalt);  8 (Ceramic tiles);  9 (Cement);  10 (Chips/terazzo/mosaic);  11 (Carpet/Mat);  12 (Polished stone/marble/granite);  99 (Other/ don’t know) |  |
|  | Categorical | Household’s main material of their wall | 1 (Cane/ palm/ trunks/ bamboo);  2 (Dirt/mud);  3 (Stone);  4 (Grass/ reed/ thatch/ sticks);  5 (Bamboo with mud);  6 (Stone with mud);  7 (Plywood);  8 (Cardboard/ plastic);  9 (Sunburn brick);  10 (Raw wood/ reused wood);  11 (Cement/ concrete);  12 (Stone with lime/ cement);  13 (Burnt bricks with lime/ cement);  14 (Finished wood planks/shingles);  15 (Cement blocks);  16 (Metal/GI or iron sheet/Tim asbestos sheet);  98 (No wall);  99 (Other/ don’t know) |  |
|  | Categorical | Household’s main source of drinking water | 1 (Piped water);  2 (Tube well/ hand pump);  3 (Dug well/ spriing water);  4 (Rain water);  5 (Water tanker/ cart);  6 (Surface);  7 (Bottled water);  8 (Filter water);  9 (From neighbors);  99 (Other/Don't Know) |  |
|  | Categorical | Household’s main type of toilet | 1 (Flush to piped sewer system);  2 (Flush to septic tank);  3 (Flush to pit latrine);  4 (Flush to somewhere else);  5 (Flush, don’t know where);  6 (Ventilated improved pit/biogas latrine);  7 (Pit latrine with slab);  8 (Pit latrine without slab/open pit);  9 (Twin pit/composing toilet);  10 (Dry toilet);  11 (Bucket toilet);  12 (Hanging toilet/hanging latrine);  13 (No facility/ bush/ open space/ field);  99 (Other/ unknown) |  |
|  | Categorical | Household has electricity? | 0 (No); 1 (Yes); 9 (Unknown) |  |
|  | Categorical | Various household assets, listed individually in notes section | 0 (No); 1 (Yes); 9 (Unknown) | Electric fan,  water pump,  CD/DVD player,  black & white television,  color television, refrigerator,  radio/transistor,  mobile telephone,  land telephone,  computer,  washing machine,  camera,  watch/clock,  pressure cooker, sewing machine, thresher,  air conditioner/room cooler,  animal drawn cart,  bicycle,  tractor/boat with motor,  car/truck,  motorcycle/scooter,  rickshaw/van,  chair,  cot/bed,  mattress,  sofa,  table,  amirah/cabinet |
|  | Categorical | Handwashing station at household? | 0 (No); 1 (Yes); 9 (Unknown) |  |
|  | Categorical | Soap available at handwashing station? | 0 (No); 1 (Yes); 3 (No handwashing station); 9 (Unknown) |  |
|  | Categorical | Water available at handwashing station? | 0 (No); 1 (Yes); 3 (No handwashing station); 9 (Unknown) |  |
|  | Categorical | Animals live at the house? | 0 (No); 1 (Yes); 9 (Unknown) |  |
| Pregnancy/  Delivery Data | Categorical | Any antenatal care received? | 0 (No antenatal appointments);  1 (At least 1 antenatal appointment);  999 (Unknown if antenatal care received) |  |
|  | Categorical | Where was antenatal care received? | 1 (Hospital);  2 (1st level facility);  3 (Outreach/satellite clinics);  4 (Doctor's chamber);  5 (At home);  6 (Auxiliary nurse midwife);  7 (Anganwadi workers);  8 (Accredited social health activists);  9 (No care);  99 (Other/ unknown); |  |
|  | Categorical | Received tetanus toxoid injection during pregnancy? | 0 (No); 1 (Yes); 9 (Unknown) |  |
|  | Continuous | Total number of tetanus toxoid injections received during pregnancy | Exact value;  99 (Unknown) |  |
|  | Categorical | Taking iron tablets/ syrups during this pregnancy? | 0 (No); 1 (Yes); 9 (Unknown) |  |
|  | Categorical | Mother smoked cigarettes during pregnancy? | 0 (No); 1 (Yes); 9 (Unknown) |  |
|  | Continuous | Maternal smoking cigarette frequency | 1 (10 or more times a day);  2 (5-9 times a day);  3 (1-4 times day);  4 (More than once a week);  5 (At least once per week);  6 (Occasionally);  7 (Did not smoke);  9 (Unknown) |  |
|  | Categorical | Mother smoked hookah during pregnancy? | 0 (No); 1 (Yes); 9 (Unknown) |  |
|  | Continuous | Maternal smoking hookah frequency | 1 (10 or more times a day);  2 (5-9 times a day);  3 (1-4 times day);  4 (More than once a week);  5 (At least once per week);  6 (Occasionally);  7 (Did not smoke);  9 (Unknown) |  |
|  | Categorical | Mother chewed tobacco during pregnancy? | 0 (No); 1 (Yes); 9 (Unknown) |  |
|  | Continuous | Maternal chewing tobacco frequency | 1 (10 or more times a day);  2 (5-9 times a day);  3 (1-4 times day);  4 (More than once a week);  5 (At least once per week);  6 (Occasionally);  7 (Did not chew tobacco);  9 (Unknown) |  |
|  | Categorical | Mother chewed betel leaf during pregnancy? | 0 (No); 1 (Yes); 9 (Unknown) |  |
|  | Continuous | Maternal betel leaf chewing frequency | 1 (10 or more times a day);  2 (5-9 times a day);  3 (1-4 times day);  4 (More than once a week);  5 (At least once per week);  6 (Occasionally);  7 (Did not chew betel leaf);  9 (Unknown) |  |
|  | Categorical | Anyone in the household smoke during pregnancy? | 0 (No); 1 (Yes); 9 (Unknown) |  |
|  | Continuous | Frequency that household member smoked in the house | 1 (10 or more times a day);  2 (5-9 times a day);  3 (1-4 times day);  4 (More than once a week);  5 (At least once per week);  6 (Occasionally);  7 (No one smoked);  9 (Unknown) |  |
|  | Continuous | Type of stove and fuel used for cooking | 1 (Gas stove using natural gas, LPG, or LNG);  2 (Gas stove using bio gas);  3 (Electric stove);  4 (Kerosene stove);  5 (Traditional mud stove using traditional biomass fuel);  6 (Traditional mud stove using charcoal);  7 (Traditional mud stove using animal residue);  8 (Improved mud stove);  9 (Other/ don’t know) |  |
|  | Continuous | Place commonly used for cooking | 1 (In the room used for living/sleeping);  2 (Separate room used as a kitchen);  3 (Separate building used as a kitchen);  4 (Verandah);  5 (Outdoors);  6 (Any corner of the house);  9 (Other/ don’t know) |  |
|  | Continuous | Ventilation used for indoor stove | 1 (Chimney);  2 (Vents or fans next to stove);  3 (Open window next to stove);  4 (Little fissure);  5 (Stove is near the door);  6 (A hole near the stove);  7 (Stove is near balcony);  8 (None);  9 (Other/ unknown) |  |
|  | Categorical | Woman worked during pregnancy? | 0 (No); 1 (Yes); 9 (Unknown) |  |
|  | Categorical | Woman performed heavy physical work during pregnancy? | 0 (No); 1 (Yes); 9 (Unknown) |  |
|  | Categorical | High grade fever during pregnancy? | 0 (No); 1 (Yes); 9 (Unknown) |  |
|  | Categorical | Excessive bleeding during pregnancy? | 0 (No); 1 (Yes); 9 (Unknown) |  |
|  | Categorical | Convulsion during pregnancy? | 0 (No); 1 (Yes); 9 (Unknown) |  |
|  | Categorical | Swelling of face or feet during pregnancy? | 0 (No); 1 (Yes); 9 (Unknown) |  |
|  | Categorical | Foul smelling vaginal discharge during pregnancy? | 0 (No); 1 (Yes); 9 (Unknown) |  |
|  | Categorical | Delivery attendant washed hands with soap before delivery? | 0 (No); 1 (Yes); 9 (Unknown) |  |
|  | Categorical | Clean delivery kit used during delivery? | 0 (No); 1 (Yes); 9 (Unknown) |  |
|  | Categorical | Color of the amniotic fluid | 0 (Clear); 1 (Not clear); 9 (Unknown) |  |
|  | Categorical | Foul smelling vaginal discharge during labor/delivery? | 0 (No); 1 (Yes); 9 (Unknown) |  |
|  | Categorical | Convulsions during labor/delivery? | 0 (No); 1 (Yes); 9 (Unknown) |  |
|  | Categorical | Excessive bleeding during labor/delivery? | 0 (No); 1 (Yes); 9 (Unknown) |  |
|  | Categorical | Retained placenta during labor/delivery? | 0 (No); 1 (Yes); 9 (Unknown) |  |
|  | Categorical | Prolonged labor? | 0 (No); 1 (Yes); 9 (Unknown) |  |
|  | Categorical | Any respiratory symptoms within 7 days before delivery? | 0 (No); 1 (Yes); 9 (Unknown) |  |
|  | Categorical | Any skin infection or pustules within 7 days before delivery? | 0 (No); 1 (Yes); 9 (Unknown) |  |
|  | Categorical | Any high fever within 7 days before delivery? | 0 (No); 1 (Yes); 9 (Unknown) |  |
|  | Categorical | Any diarrhea within 7 days before delivery? | 0 (No); 1 (Yes); 9 (Unknown) |  |
|  | Categorical | Any household member ill within 7 days before delivery? | 0 (No); 1 (Yes); 9 (Unknown) |  |
