# Supplementary material for: Mortality risk associated with clinical signs of possible serious bacterial infection (PSBI) in young infants in Africa and Asia: protocol for a secondary pooled analysis
Source: BMJ Open. 2025 Jun 24;15(6):e097135. doi: 10.1136/bmjopen-2024-097135 (PMC12198825; doi:10.1136/bmjopen-2024-097135)
Supplement: Supplementary file 2 [file bmjopen-2024-097135.reviewer_comments.pdf]

PEER REVIEW HISTORY

BMJ Open publishes all reviews undertaken for accepted manuscripts. Reviewers are asked to complete a checklist review form and are provided with free text boxes to elaborate on their assessment. These free text comments are reproduced below.

ARTICLE DETAILS

Title (Provisional)

Mortality risk associated with clinical signs of possible serious bacterial infection (PSBI) in young infants in Africa and Asia: protocol for a secondary pooled analysis

Authors

Darmstadt, Gary L; Bhamidi, Vaishnavi; Adhikari, Khusbu; Marić, Ivana; Islam, Shahidul; Qazi, Shamim; Ahmed, Saifuddin; Tshefu Kitoto, Antoinette; Esamai, Fabian; Ayede, Adejumoke Idowu; Adejuyigbe, Ebunoluwa A.; Wammanda, Robinson D; Saha, Samir; Nisar, Yasir Bin

---

VERSION 1 - REVIEW

**Reviewer**	**1**
**Name**	**Martin, Blake**
**Affiliation**	**Children's Hospital Colorado, Pediatrics**
**Date**	**05-Feb-2025**
**COI**	**None**

Thank you for the opportunity to review the manuscript entitled "Mortality risk associated with clinical signs of possible serious bacterial infection (PSBI) in young infants in Africa and Asia: protocol for a secondary pooled analysis" by Darmstadt and colleagues. In this study protocol, the authors detail their plan for a pooled analysis of infants (0-60 days) evaluated for signs of possible serious bacterial infection (PSBI) from within two large studies performed in Asia and Africa. The authors intend to conduct three categories of analysis using this retrospective data: case fatality, Cox regression, and machine learning (ML, using multiple commonly used and powerful ML algorithms). The authors will analyze the varying importance and predictive ability of seven PSBI clinical indicators with regard to mortality. The goal is to determine how to best leverage these clinical indicators to enable local health workers to gauge infant mortality risk and inform hospital referral, thus contributing to the World Health Organization's Integrated Management of Childhood Illness (IMCI) strategy.

Overall, the protocol is well written, and the study has significant potential impact. By improving the strategy by which community health workers identify children with PSBI, this project could directly lead to early referral and antibiotics for the highest risk children while minimizing unnecessary resource allocation toward low-risk infants that can be safely

managed as outpatients. See below for a few points / questions which I would like the authors to address, and which I believe would improve the clarity of this important work.

1. Do we know anything about the interrater reliability of these seven signs of PSBI? If so, inclusion in the methods section would be appropriate as this would have implications for the utility of the ANISA and AFRINEST data and could impact implementation of any tools developed through this study.

2. *"Young infants whose information on signs of PSBI at either of the two visits immediately preceding the outcome is not available will be excluded from the analysis."* Do the authors have a sense for how often any PSBI indicators are absent from visit documentation? The number of deaths in both studies is not extremely high. As such, exclusion of many these cases from the analysis could significantly impact the authors' ability to conduct their planned analyses. If this is the case, it might be helpful to perform a sensitivity analysis in which you impute missing PSBI indicators as normal/absent (or carry forward the last available value for each PSBI) and then include these cases. However, if omission of PSBI from visits is rare, then the authors' original approach would be reasonable. Along these lines, how often were children hospitalized in these two studies? When they were hospitalized, did the community health workers still visit with the patients and document PSBI indicators? If not, omission of patients with missing PSBI indicators in the two visits before mortality could lead to the bias of excluding children who were hospitalized for their illness.

3. Though the authors briefly touch on this, there should be more explicit discussion of how the authors will handle the variation in antibiotic treatment between the patients in the study. As one study was a 3-arm community-based antibiotic treatment trial and the other study was an observational study in which antibiotics were prescribed per local practice, there could be significant differences in patient outcomes between the two groups with respect to mortality. A bit more detail on how the authors control for this would be helpful.

4. *"The period of risk for mortality will be defined as extending for 7 days after finding a sign of PSBI; a sensitivity analysis will be performed using a 14-day period of risk for mortality."* Is there data available to suggest that if death occurs it will typically occur in this window after a PSBI is identified? If so, it should be cited. If not, the authors should explain the physiologic rationale for using these specific time periods.

5. *"The presence/absence of PSBI signs recorded over multiple visits, as well as available clinical and treatment data will be used as input features"* This sentence suggests that there will be many predictors included within the machine learning (ML) models other than the seven indicators of PSBI. It would be helpful to get a better sense for what types and how many '*clinical and treatment data'* predictors will be included within the models. If there is a large amount of these other predictors, then the predictive utility of the models may become relatively detached from the seven PSBI indicator the authors initially sought to better understand. The authors should identify how many additional predictors they expect to include in these models and expound on the types of variables they will include.

6. While the ML analysis planned is robust and well thought out, it remains unclear how the authors foresee these models being applied / implemented in the community. If the goal is to provide a new tool to community health workers to better assess PSBI and mortality risk, then it will need to be in the form of a tool that such a worker can easily understand and utilize. Even a brief description in the intro or methods for how the authors foresee these models being used in the future would help ground the work and enable the reader to better appreciate the potential clinical impact.

**Reviewer**	**2**
**Name**	**Gladstone, Beryl Primrose**
**Affiliation**	**University Hospital Tuebingen, Infectious Diseases**
**Date**	**03-Mar-2025**
**COI**	**None**

The authors present a well-written study protocol to analyse an important aspect of serious bacterial infection signs impacting young infant deaths. The authors intend to use machine learning to dissect out the importance of each of the 7 signs recommended by WHO, in addition to the conservative ways of analysis, which is very interesting and novel.

I have some comments and questions:

1. The reason cited for the exclusion of the SATT Pakistan trial ("In page 7 - lines 19-31, the SATT Pakistan trial utilised community health workers (CHWs) to assess young infants for signs of PSBI in the community…. Since our study is aimed to assess risk of mortality associated with signs of PSBI identified by CHWs and not nurses, and thus to inform IMCI, the SATT Pakistan study was excluded.") is not convincing because it is similar to AFRINEST in the identification and management part (in table 1, it is clear that the AFFRINEST trial also identified PBSI signs by CHW and referred the infants to nurses for further assessment and management ("CHWs in DRC and Kenya and CHEWs in Nigeria visited infants at home on days 0, 2, 6, 13, 20, 27, 34, 41, 48, 59. They identified sick young infants with PSBI signs and referred them to registered nurses at the health centres for further assessment and management.")

2. The AFRINEST is an interventional trial (RCT) and all the arms had a trial protocol to follow and assigned to various antibiotic treatments to prove its equivalence, which epidemiologically is a unique scenario – not to be compared with an observational study with standard of care. This is also reflected in the mortality rate of AFRINEST which is 6 per 1000 infants, much lower as compared to 26/1000 infants in ANISA. Looking at the infant mortality rates, it does not seem to be that Asian countries have a higher infant mortality rate than African setting. So it would be necessary to keep in mind that the case fatality rates from the pooled data may not reflect the true indices in Africa/Asia – knowing that the better management of cases in AFRINEST trial would underestimate the actual risk of

mortality associated in a normal setting. Relative importance of each of the 7 signs may not be affected; however, external validity of the mortality risk for the individual signs are at stake and should be discussed in the protocol and pointed out while presenting the results later.

3. The first two of the three analytical procedures are just two steps of the standard conventional statistics approach - the first one is the basic step - a simple unadjusted look at data – providing unadjusted estimates of proportion (risk of mortality), rate per time (per infant-periods – survival analysis); the second one is an adjusted advanced look at the data – Cox regression is being used to arrive at the adjusted hazard estimates with the same survival data. The machine learning aspect helps to calculate the risks in a more complex manner. However, the last step uses Cox proportional hazards model to bring out the meaningful time to event.

In this context, I would rather suggest to mention the analysis as comparison of 2 analytical approaches - the first 2 analytical procedures as a conventional approach and the third one as the machine learning approach.

4. In the methods (page 10 – line 22), it says “Case fatality will be calculated as the ratio of the number of young infant deaths among all young infants with a given clinical sign and expressed per 1000 infants. It is a bit vague what the ratio of what and what? – does the ratio refer to infant deaths among infants with a specific sign to 1000 live infants with a specific sign? or infant deaths among patients with a specific sign to 1000 infants without the specific sign? Could you please clarify in the text?

5. I would suggest that a sensitivity analysis is carried out at the study and centre level to understand the variability and validity.

VERSION 1 - AUTHOR RESPONSE

Comments from Reviewer: 1

Dr. Blake Martin, Children's Hospital Colorado

Do we know anything about the interrater reliability of these seven signs of PSBI? If so,

inclusion in the methods section would be appropriate as this would have implications for the utility of the ANISA and AFRINEST data and could impact implementation of any tools

developed through this study.

Response: The data were not coded for identification of individual community health workers (CHWs), and thus, we cannot calculate interrater reliability of CHW assessment. Within the ANISA dataset, we have compared the identification of signs by CHWs with the identification of signs by physicians who saw the young infants after referral by the CHWs. These results will be published separately in a manuscript currently under review at

eClinicalMedicine. Previously, agreement (kappa) has been reported by Baqui et al. (Baqui AH, … Darmstadt GL. Community-based validation of assessment of newborn illnesses by trained community health workers in Sylhet district of Bangladesh. Trop Med Int Health. 2009 Dec;14(12):1448-56. doi: 10.1111/j.1365-3156.2009.02397.x) and Darmstadt et al. (Darmstadt GL, et al. Validation of a clinical algorithm to identify neonates with severe illness during routine household visits in rural Bangladesh. Arch Dis Child. 2011;96:1140-6. doi: 10.1136/archdischild-2011-300591) between CHW and physician assessments, and agreement has been examined between village-based community health volunteers and more highly trained, paid facility-based CHWs in Nepal (Khanal S, et al. Community health workers can identify and manage possible infections in neonates and young infants: MINI—a model from Nepal. J Health Popul Nutr. 2011; 29(3):255–64), but interrater reliability among CHWs has not been reported.

A statement about our inability to calculate interrater reliability among CHW assessments has been added to the Methods section, subsection Data analysis, as follows: "Due to lack of information on the individual identification of CHW assessments, interrater reliability cannot be determined; however, CHWs were regularly standardised for assessment of clinical signs, as described previously."

2. "Young infants whose information on signs of PSBI at either of the two visits immediately preceding the outcome is not available will be excluded from the analysis." Do the authors have a sense for how often any PSBI indicators are absent from visit documentation? The number of deaths in both studies is not extremely high. As such, exclusion of many these cases from the analysis could significantly impact the authors' ability to conduct their planned analyses. If this is the case, it might be helpful to perform a sensitivity analysis in which you impute missing PSBI indicators as normal/absent (or carry forward the last available value for each PSBI) and then include these cases. However, if omission of PSBI from visits is rare, then the authors' original approach would be reasonable. Along these lines, how often were children hospitalized in these two studies? When they were hospitalized, did the community health workers still visit with the patients and document PSBI indicators? If not, omission of patients with missing PSBI indicators in the two visits before mortality could lead to the bias of excluding children who were hospitalized for their illness.

Response: It is likely that we mis-stated our description of the case fatality (CFR) analysis. The infants will be not excluded but their unknown PSBI status at the "not visit period" will

be censored. We explained below (and added the italicized text to the Methods section Case fatality analysis subsection) in response to another reviewer's comment, "Case fatality rate will be calculated by sign or a combination of signs as the percentage of young infants who die within 7 days after showing that sign or a combination of signs of PSBI, divided by the total number of young infants who had shown that same sign or combination of signs during their previous (reference) visit. We specified a 7-day window period to avoid the risk of misclassification or recall bias when no visit data were available for the extended period." So, all children who were ever visited by CHWs and reported a PSBI sign will be included in the denominator of CFR calculations. In case of death (numerator) occurring within 7 days, the cause of death will be attributed to PSBI and children will be included in the numerator irrespective of the children's subsequent visit status. However, if the children did not receive any subsequent visit and no information was available about the PSBI status for more than 7 days before death during the observation window, we will exclude those children from the numerator because we consider that it may introduce (overestimation) bias because there is a possibility that children may have died from another (unknown) cause and for uncertainty about the cause of death as these PSBI signs are considered as acute conditions.

When infants were hospitalized, CHWs did not continue to visit the patients and document PSBI signs while the infants were in the hospital; however, CHWs did continue to assess these infants according to the standard visit schedule after discharge from the hospised, and these infants will be retained in the analysis. In ANISA, 4% of infants were hospitalised, while in AFRINEST, 5% of infants were hospitalised. This information (italicised text) has been added to the Methods in the Analysis subsection.

3. Though the authors briefly touch on this, there should be more explicit discussion of how the authors will handle the variation in antibiotic treatment between the patients in the study. As one study was a 3-arm community-based antibiotic treatment trial and the other study was an observational study in which antibiotics were prescribed per local practice, there could be significant differences in patient outcomes between the two groups with respect to mortality. A bit more detail on how the authors control for this would be helpful.

Response: In ANISA, physician recommendation based on their assessment of young infants was categorized as referral/admission, refusal of referral/admission, or home treatment recommended; thus, it was possible to examine the association of signs of PSBI with mortality by place of treatment. However, no additional information was recorded on how infants were managed, and we cannot adjust for this in the analysis.

We have added this statement to the Methods section, Data analysis subsection: "A sensitivity analysis will be carried out at the study (ANISA and AFRINEST) and site level."

4. "The period of risk for mortality will be defined as extending for 7 days after finding a sign of PSBI; a sensitivity analysis will be performed using a 14-day period of risk for mortality."

Is there data available to suggest that if death occurs it will typically occur in this window after a PSBI is identified? If so, it should be cited. If not, the authors should explain the physiologic rationale for using these specific time periods.

Response: Among all infants in the ANISA study, 72% of deaths occurred in the first week and 59% of deaths took place within 3 days of birth; this data has been summarized in another manuscript on causes and risk factors for mortality in ANISA that is under review. We also conducted a sensitivity analysis of association of signs of PSBI with 7-day vs 14-day periods of risk for mortality in the ANISA study, and found no substantial differences; this data has been published (Darmstadt GL, et al. Association of clinical signs of possible serious bacterial infections identified by community health workers with mortality of young infants in South Asia: a prospective, observational cohort study. EClinicalMedicine. 2025 Jan 18;80:103070. doi: 10.1016/j.eclinm.2025.103070).

The following sentence has been added to the Methods section under the subsection, Cox regression analysis: "We showed previously that for the ANISA data, results were similar for these two periods of risk for mortality."

5. "The presence/absence of PSBI signs recorded over multiple visits, as well as available clinical and treatment data will be used as input features" This sentence suggests that there will be many predictors included within the machine learning (ML) models other than the seven indicators of PSBI. It would be helpful to get a better sense for what types and how many 'clinical and treatment data' predictors will be included within the models. If there is a large amount of these other predictors, then the predictive utility of the models may become relatively detached from the seven PSBI indicator the authors initially sought to better understand. The authors should identify how many additional predictors they expect to include in these models and expound on the types of variables they will include.

Response: The following sentence has been added, following the sentence above: "See supplemental table S1 for a preliminary list of available predictors that are common to the ANISA and AFRINEST studies (which includes all predictors in AFRINEST), and supplemental table S2 of predictors available in the ANISA dataset."

6- While the ML analysis planned is robust and well thought out, it remains unclear how the authors foresee these models being applied / implemented in the community. If the goal is to provide a new tool to community health workers to better assess PSBI and mortality risk, then it will need to be in the form of a tool that such a worker can easily understand and utilize. Even a brief description in the intro or methods for how the authors foresee these models being used in the future would help ground the work and enable the reader to better appreciate the potential clinical impact.

Response: We hypothesize that the ML methods will enable us to gain additional insights into combinations of signs of PSBI, and potentially other factors that identify young infants with particularly heightened risk for mortality. This information will be taken into account in considering modifications to Integrated Management of Childhood Illness. We will also consider the development of an app – starting with fast breathing in the first week after birth – to be used by CHWs to guide the management of infants based on information that is useful in predicting risk for mortality.

The following test has been added to the end of the Methods section: “Results from these analyses will enable us to gain additional insights, particularly from ML analysis, into combinations of signs of PSBI, and potentially other factors that identify young infants with particularly heightened risk for mortality. This information will be taken into account in considering modifications to IMCI for young infants. We will also consider the development of an app – starting, for example, with fast breathing in the first week after birth – to be used by CHWs to guide the management of infants based on information that is useful in predicting risk for mortality.”

Reviewer: 2

Dr. Beryl Primrose Gladstone, University Hospital Tuebingen

1. The reason cited for the exclusion of the SATT Pakistan trial (“In page 7 - lines 19-31, the SATT Pakistan trial utilised community health workers (CHWs) to assess young infants for signs of PSBI in the community…. Since our study is aimed to assess risk of mortality associated with signs of PSBI identified by CHWs and not nurses, and thus to inform IMCI, the SATT Pakistan study was excluded.”) is not convincing because it is similar to AFRINEST in the identification and management part (in table 1, it is clear that the AFFRINEST trial also identified PBSI signs by CHW and referred the infants to nurses for further assessment and management (“CHWs in DRC and Kenya and CHEWs in Nigeria visited infants at home on days 0, 2, 6, 13, 20, 27, 34, 41, 48, 59. They identified sick young infants with PSBI signs and referred them to registered nurses at the health centres for further assessment and management.”)

Response: The additional reason that SATT Pakistan was not included is that we contacted the authors of the study and they were unable to provide data on assessments across the entire young infant study on all young infants.

The following text (in italics) was added in the Methods section, Study population subsection: “Since our study is aimed to assess risk of mortality associated with any sign of PSBI, alone or in combination, identified by CHWs and not physicians and because data on CHW assessments across the entire young infant period were not available on infants

enrolled in the SATT Pakistan study (as determined through discussion with the study principal investigators), this study was excluded.

2. The AFRINEST is an interventional trial (RCT) and all the arms had a trial protocol to follow and assigned to various antibiotic treatments to prove its equivalence, which epidemiologically is a unique scenario – not to be compared with an observational study with standard of care. This is also reflected in the mortality rate of AFRINEST which is 6 per 1000 infants, much lower as compared to 26/1000 infants in ANISA. Looking at the infant mortality rates, it does not seem to be that Asian countries have a higher infant mortality rate than African setting. So it would be necessary to keep in mind that the case fatality rates from the pooled data may not reflect the true indices in Africa/Asia – knowing that the better management of cases in AFRINEST trial would underestimate the actual risk of mortality associated in a normal setting. Relative importance of each of the 7 signs may not be affected; however, external validity of the mortality risk for the individual signs are at stake and should be discussed in the protocol and pointed out while presenting the results later.

Response: The AMANHI study that included many sites in common with AFRINEST (Democratic Republic of Congo, Kenya) and ANISA (Sylhet, Bangladesh; Karachi and Matiari, Pakistan) showed that the neonatal mortality rate was approximately 2-fold higher for infants in South Asia (43·0 per 1000 livebirths) compared to sub-Saharan Africa (20·1 per 1000 livebirths) (Alliance for Maternal and Newborn Health Improvement (AMANHI) mortality study group. Population-based rates, timing, and causes of maternal deaths, stillbirths, and neonatal deaths in south Asia and sub-Saharan Africa: a multi-country prospective cohort study. Lancet Glob Health. 2018;6(12):e1297-e1308. doi: 10.1016/S2214-109X(18)30385-1). As noted by the reviewer, however, the overall mortality rate for the AFRINEST study (9.2 deaths in infants <60 days of age per 1000 livebirths) was lower than what AMANHI would suggest, although the rate in ANISA [overall young infant mortality rate (days 0-<60 days) 43.0 per 1,000 live births] was consistent with AMANHI (mortality rates in ANISA are reported in another manuscript under review). Thus, we acknowledge that the case fatality rates from the pooled data may not reflect the true picture in Africa.

Please note that we calculated the mortality rate in AFRINEST as follows: In AFRINEST, there were 86621 births for follow up by CHWs. Of these, 196 died before the CHWs could make their first visit. Additionally, 833 infants received only one visit during the young infant period (less than 2 months old) and were alive so we don't know their vital status. Out of 84759 infants who were followed up for 2 months by CHWs, we recorded 587 deaths. This results in a mortality rate of 6.9 deaths per 1,000 live births during the first two months. If we include the 196 deaths in both the numerator and the denominator, while excluding the 833 cases, the adjusted rate is 9.2 deaths per 1,000 live births in infants under 2 months of age.

Reference: Puri D, et al. PlosOne 2021 (https://pubmed.ncbi.nlm.nih.gov/33626090/).

The following text was added to the Methods section, Study population subsection: “It is notable that the young infant mortality rate in the AFRINEST study (9.2 deaths in infants <60 days of age per 1000 livebirths) was substantially lower than the neonatal mortality rate for sub-Saharan Africa reported by AMANHI (20.1 per 1000 livebirths), which may reflect the treatment provided to infants in the AFRINEST study and may limit generalisability of the results of our analysis.”

While treatment was more regimented for infants with signs of PSBI in AFRINEST than ANISA, nevertheless, treatment according to the local standard of care was provided to infants in ANISA. We only have data on the initial physician assessment and treatment recommendation in ANISA, but do not have detail on the specifics of the treatment regimen, for example, we do not know which antibiotics were administered. We will examine the risk for mortality associated with signs of PSBI separately and combined for AFRINEST and ANISA.

The following text was added to the Methods section, Data analysis subsection: “A sensitivity analysis of mortality risk will be carried out at the study (ANISA and AFRINEST) and site level.”

3. The first two of the three analytical procedures are just two steps of the standard conventional statistics approach - the first one is the basic step - a simple unadjusted look at data – providing unadjusted estimates of proportion (risk of mortality), rate per time (per infant-periods – survival analysis); the second one is an adjusted advanced look at the data – Cox regression is being used to arrive at the adjusted hazard estimates with the same survival data. The machine learning aspect helps to calculate the risks in a more complex manner. However, the last step uses Cox proportional hazards model to bring out the meaningful time to event.

In this context, I would rather suggest to mention the analysis as comparison of 2 analytical approaches - the first 2 analytical procedures as a conventional approach and the third one as the machine learning approach.

Response: We acknowledge the reviewer’s perspective, but prefer to maintain these as three analytic approaches, as Cox regression is time varying and thus differs in this significant way from the case fatality analysis, which does not take into account the varying time from exposure to outcome.

4. In the methods (page 10 – line 22), it says “Case fatality will be calculated as the ratio of the number of young infant deaths among all young infants with a given clinical sign and expressed per 1000 infants. It is a bit vague what the ratio of what and what? – does the ratio refer to infant deaths among infants with a specific sign to 1000 live infants with a specific sign? or infant deaths among patients with a specific sign to 1000 infants without the specific sign? Could you please clarify in the text?

Response: We clarified this text as follows: “Case fatality rate will be calculated by sign or combination of signs as the percentage of young infants who die within 7 days after showing that sign or combination of signs of PSBI, divided by the total number of young infants who had shown that same sign or combination of signs during their previous (reference) visit. We specified a 7-day window period to avoid the risk of misclassification or recall bias when no visit data were available for the extended period.”

5. I would suggest that a sensitivity analysis is carried out at the study and centre level to understand the variability and validity.

Response: This will be done. Mention of this has been added to the Methods section, Data analysis subsection: “A sensitivity analysis for mortality risk will be carried out at the study (ANISA and AFRINEST) and site level.”

Reviewer: 1 competing interests.: Not applicable

Reviewer: 2 competing interests.: Not applicable

VERSION 2 - REVIEW

**Reviewer**	**1**
**Name**	**Martin, Blake**
**Affiliation**	**Children's Hospital Colorado, Pediatrics**
**Date**	**28-Apr-2025**
**COI**	

I thank the authors for addressing all of my concerns and clarifying my questions. I have no further requests. Best of luck in your study.

**Reviewer**	**2**
**Name**	**Gladstone, Beryl Primrose**
**Affiliation**	**University Hospital Tuebingen, Infectious Diseases**
**Date**	**23-Apr-2025**
**COI**	

I commend the authors for the well planned study and best wishes for the research work!
